# PI3K Plays an Essential Role in Planarian Regeneration and Tissue Maintenance

**DOI:** 10.3389/fcell.2021.649656

**Published:** 2021-08-06

**Authors:** Hanxue Zheng, Hongbo Liu, Qian Xu, Wenjun Wang, Linfeng Li, Gang Ye, Xiaomin Wen, Fulin Chen, Yuan Yu

**Affiliations:** ^1^Laboratory of Tissue Engineering, College of Life Sciences, Northwest University, Xi’an, China; ^2^Provincial Key Laboratory of Biotechnology of Shaanxi, Northwest University, Xi’an, China; ^3^Key Laboratory of Resource Biology and Biotechnology in Western China, Ministry of Education, School of Medicine, Northwest University, Xi’an, China

**Keywords:** PI3K, planarian, regeneration, stem cell, cellular response

## Abstract

Phosphatidylinositol 3-kinase (PI3K) signaling plays a central role in various biological processes, and its abnormality leads to a broad spectrum of human diseases, such as cancer, fibrosis, and immunological disorders. However, the mechanisms by which PI3K signaling regulates the behavior of stem cells during regeneration are poorly understood. Planarian flatworms possess abundant adult stem cells (called neoblasts) allowing them to develop remarkable regenerative capabilities, thus the animals represent an ideal model for studying stem cells and regenerative medicine *in vivo*. In this study, the spatiotemporal expression pattern of *Djpi3k*, a PI3K ortholog in the planarian *Dugesia japonica*, was investigated and suggests its potential role in wound response and tissue regeneration. A loss-of-function study was conducted using small molecules and RNA interference technique, providing evidence that PI3K signaling is required for blastema regrowth and cilia maintenance during planarian regeneration and homeostasis. Interestingly, the mitotic and apoptotic responses to amputation are substantially abated in PI3K inhibitor-treated regenerating animals, while knockdown of *Djpi3k* alleviates the mitotic response and postpones the peak of apoptotic cell death, which may contribute to the varying degrees of regenerative defects induced by the pharmacological and genetic approaches. These observations reveal novel roles for PI3K signaling in the regulation of the cellular responses to amputation during planarian regeneration and provide insights for investigating the disease-related genes in the regeneration-competent organism *in vivo*.

## Introduction

Phosphatidylinositol 3-kinase is a ubiquitous protein kinase that participates in multiple cellular processes, including cell proliferation, apoptosis, differentiation, and migration (Madsen, 2020). The catalytic subunit of the kinase converts phosphatidylinositol 4,5-biphosphate into phosphatidylinositol 3,4,5-triphosphate, which recruits phosphoinositide-dependent protein kinase-1 to phosphorylates and activates its downstream effector Akt serine/threonine kinase (Chen et al., 2013). It is now well established from a variety of studies that PI3K plays a crucial role in cancer, diabetes, and neurodegenerative diseases (Thorpe et al., 2015; Huang et al., 2018; Xu et al., 2020). Intriguingly, it has been found that chemical inhibitors against PI3K catalytic subunit, such as LY294002 and A66, either alone or in combination with other therapeutics, are effectively used in preclinical studies for the treatment of related diseases (Courtney et al., 2010; Jamieson et al., 2011).

Animal regeneration is an essential biological phenomenon, and its abnormalities are closely related to the occurrence of many diseases (Scimone et al., 2020). Therefore, it is of great significance to the investigation of regeneration processes and regulation mechanisms. In recent years, there has been a shred of increasing evidence suggesting the important role of the PI3K pathway in animal regeneration. In hydra, PI3K participates in the regulation of head formation *via* activating the early head-specific genes (Manuel et al., 2006). Biliary epithelial cells (BECs) are facultative liver stem cells that give rise to hepatocytes following injury, the positive role of the PI3K pathway in BEC-driven liver regeneration has been demonstrated in the zebrafish hepatocyte ablation model (Jung et al., 2020). For amphibian limb regeneration, PI3K facilitates the formation of limb blastema by controlling the reentry of mesodermal cells into the cell cycle in the *Xenopus laevis* froglet (Suzuki et al., 2007); several components of the PI3K pathway have been identified to be closely associated with *Cynops orientalis* limb regeneration by using an iTRAQ-based proteomic approach (Tang et al., 2017). In mice, PI3K activation promotes sensory axon regeneration (Saijilafu et al., 2013) and hepatic regenerative response (Jackson et al., 2008). Despite extensive research on PI3K, the mechanisms by which PI3K signaling regulates the regenerative process have yet to be thoroughly elucidated.

The planarian *Dugesia japonica*, a species of triclad flatworm that inhabits the freshwater bodies of East Asia, has long been an excellent model organism for the study of regeneration (Cebrià et al., 2002; Yazawa et al., 2009; Yujia et al., 2019). The remarkable regenerative ability of planarians derives from an abundance of adult stem cells, also known as neoblasts, that are distributed throughout the mesenchyme and account for 20–30% of all planarian cells (Wagner et al., 2011). During normal tissue turnover and regeneration after injury, neoblasts serve as the only mitotically active cells in the adult to give rise to all types of planarian cells, including neurons, muscle, and epidermal cells (Reddien and Sánchez Alvarado, 2004; Hayashi et al., 2006). Therefore, planarian *Dugesia japonica* represents an emerging model for unraveling the mechanisms of PI3K signaling involved in regeneration events.

In this study, we identified and characterized a planarian PI3K-like gene, *Djpi3k.* The expression pattern of *Djpi3k* was profiled in intact and regenerating planarians. Functional analysis using small molecular inhibitors LY294002/A66 or RNA interference (RNAi) technique showed that PI3K signaling is required for planarian regeneration and homeostasis. We also found that inhibition of PI3K signaling attenuates the mitotic and apoptotic responses to amputation. Our work reveals the role and mechanism of PI3K signaling in the regulation of planarian regeneration.

## Materials and Methods

### Planarian Culture

A clonal strain of the planarian *Dugesia japonica* was used in all experiments (Wang et al., 2021). The colony was maintained in autoclaved stream water in the dark at 20°C. Planarians 6–8 mm in length were starved for at least one week. Two regenerating fragments were obtained *via* transverse amputation at the anterior of the pharynx.

### Bioinformatic Analysis

DNAMAN software (7.0) was used to analyze the obtained cDNA and deduced the amino acid sequence of *Djpi3k*. The conserved domains of Djpi3k protein were predicted using the NCBI Conserved Domain Database (Lu et al., 2020) and the SMART database (Letunic et al., 2021). The ProtParam online tool^1^ was used to calculate the molecular weight and isoelectric point (pI) of Djpi3k protein. Through the neighbor-joining (NJ) method, a phylogenetic tree was constructed *via* MEGA software (7.0), and further annotation was conducted by the Evolview tool using a Pfam database (He et al., 2016).

### Reverse Transcription and Quantitative Real-Time PCR Analysis

At 0 h, 4 h, 8 h, 12 h, 1 days, 3 days, 5 days, 7 days post-amputation, head- and tail-regenerating tissues were harvested and grinded using a homogenizer, respectively. For each replicate, 5–10 planarians were homogenized together, and total RNA was isolated using RNAiso Plus reagent (Takara, China) according to the standard protocol. Next, cDNA was synthesized from 1 μg of total RNA using Transcriptor First Strand cDNA Synthesis Kit (Roche, Switzerland). The qRT-PCR analysis was performed using SYBR Premix Ex Taq (TaKaRa, China) and a CFX96^TM^ Real-Time PCR Detection System (Bio-Rad, United States) with the following parameters: 95°C for 10 s; followed by 40 cycles at 95°C for 10 s, 60°C for 10 s, and 72°C for 10 s. The β*-actin* housekeeping gene was selected as an internal reference. The relative gene expression of each gene was calculated using the 2^–ΔΔ*CT*^ method. PCR primers used in this study were shown in Supplementary Table 1.

### Treatment With Chemical Inhibitors

The PI3K-specific inhibitor LY294002 (Abmole Bioscience, United States) or A66 (Abmole Bioscience, United States) was dissolved in DMSO and used at a series of gradient concentrations. Planarians were allowed to regenerate in water supplemented with LY294002 or A66 immediately after amputation until the indicated period of regeneration. For homeostasis experiments, intact animals were maintained in water supplemented with 30 μM LY294002 for 20 days.

### RNAi Experiment

RNAi was conducted by oral delivery of bacterially expressed dsRNA as described previously (Newmark et al., 2003; Zeng et al., 2013). Briefly, two fragments against the Class I PI3K accessory and catalytic domains of the *Djpi3k* gene were introduced into the L4440 vector (BioVector NTCC, China) between two convergent T7 promoters. The RNAi vectors were transformed into an RNaseIII-deficient *E. coli* strain HT115 (BioVector NTCC, China) to generate dsRNA by IPTG induction (0.4 mM, Amresco, United States). Then animals were fed four rounds with a mixture of the dsRNA-expressing bacteria and liver paste. Animals fed with HT115 bacteria carrying a fragment encoding EGFP served as a negative control. Three days after the last feeding, the RNAi efficiency was examined by whole-mount *in* s*itu* hybridization (WISH) and qRT-PCR.

### Whole-Mount *in situ* Hybridization

Whole-mount *in situ* hybridization was performed using a slightly modified protocol previously described (Pearson et al., 2009). Digoxigenin-labeled antisense RNA probes were synthesized using the *in vitro* RNA labeling kit (Roche, Germany). Prior to 5% N-Acetyl Cysteine treatment and 4% paraformaldehyde fixation, planarians were treated with 2% HCl to kill and remove mucus for 5 min. After bleaching and rehydration, specimens were treated with proteinase K (10 mg/mL, Merck, Germany) and fixed in 4% paraformaldehyde at room temperature. Hybridizations with the labeled probe in the concentration of 1 ng/μL were performed at 56°C for 16 h after pre-hybridization. Next, specimens were washed with preheated (to 56°C) gradient solutions. Blocking and incubation of the anti-Digoxigenin-AP antibody (1:5,000, Roche, Germany) were done overnight at 4°C. Following 5-Bromo-4-chloro-3-indolyl phosphate and nitroblue tetrazolium (Roche, Germany) development to detect the Dig-labeled riboprobe.

### Whole-Mount Immunostaining

The planarians were sacrificed in ice-cold 2% HCl for 2–5 min and fixed in 4% paraformaldehyde, then rinsed in 4°C methanol for 1 h and bleached in methanol containing 6% H_2_O_2_ overnight. After rehydration, specimens were blocked in 0.6% BSA and 0.45% fish gelatin diluted with PBSTx for 1 h at room temperature and incubated sequentially in primary antibody concentrations were used as follows: anti-phospho-Histone H3 (anti-H3P, 1:250, R&D Systems, United States); anti-Acetylated Tubulin (anti-Ac-Tubulin, 1:500, Sigma, United States); anti-SYNORF1 (1:50, Developmental Studies Hybridoma Bank, United States). Secondary antibody concentrations were: goat-anti-mouse IgG (1:200, Millipore, United States), and goat anti-rabbit IgG (Alexa Fluor488) (1:1,000, Abcam, United Kingdom). The signals were photographed and fluorescent images were captured and analyzed with LEICA SP8 confocal microscope and ImageJ software.

### TUNEL Assay

A whole-mount TUNEL assay using the planarians was performed as described previously (Pellettieri et al., 2010). After fixation, the planarians were permeabilized in 1% SDS for 20 min and bleached overnight in bleached in methanol containing 6% H_2_O_2_ overnight. Next, specimens were incubated with the terminal transferase enzyme diluted in label solution (Roche, Germany) for 4 h at 37°C, and then rinsed in PBSTB (PBST with 0.25% BSA), and these animals were incubated in anti–digoxigenin-rhodamine diluted in blocking solution for 4 h at room temperature. DAPI was used to stain nuclei. The signals were photographed and fluorescent images were captured and analyzed with LEICA SP8 confocal microscope and ImageJ software.

### Measurements of Planaria and Image Processing

Planarian live and WISH images were acquired with Canon EOS 600D and OLYMPUS SZX10 microscopes, respectively. Z-stacks of fluorescently labeled samples were acquired with a LEICA SP8 confocal microscope and processed with the ImageJ package Fiji. For quantification of H3P-positive or TUNEL-positive cells, stacks with equal numbers of z-sections were taken and cells were counted in the indicated areas using ImageJ package Fiji. To quantify cilia density, the fluorescence intensity of the Ac-tubulin signal was measured and normalized to DAPI fluorescence. To assess blastema formation, the length of the regenerated blastema was measured using ImageJ package Fiji and normalized to the whole-body length of the trunk. The ratio of blastema/whole-body length of small molecules treated animals or *Djpi3k*(RNAi) animals were compared with control animals at each time point.

### Statistical Analysis

Values were expressed as mean ± SEM. The statistical differences were calculated using the Student’s *t*-test in SPSS 21.0 software. Statistical significance is indicated as follows: ^∗^*P* < 0.05, ^∗∗^*P* < 0.01, ^∗∗∗^*P* < 0.001, and ns, not significant.

## Results

### Characterization of a PI3K-Like Gene in Planarian

A planarian PI3K-like gene, *Djpi3k*, was cloned from *Dugesia japonica* and contains an ORF of 3,075 bp. The predicted Djpi3k protein is composed of 1,024 amino acid residues and shares conserved functional domains with the catalytic subunit of Class I PI3K, including p85-binding domain, Ras-binding domain, C2 domain, PI3K accessory, and catalytic domains (Supplementary Figure 1). The molecular weight of the predicted Djpi3k protein was estimated to be 256.21 kDa, and the pI was estimated at 4.98.

**FIGURE 1:**
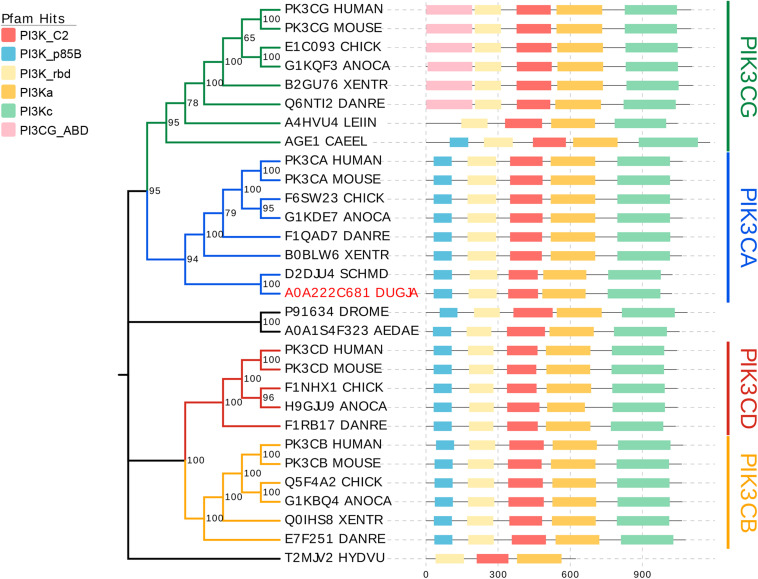
Phylogenetic analysis of catalytic PI3Ks subunits. All selected genes encoding the catalytic subunits of Class I PI3K are mainly categorized into four clusters corresponding to PIK3CA (blue), PIK3CB (yellow), PIK3CD (red), and PIK3CG (green). Domain annotations for each sequence were determined using the Pfam database (Mistry et al., 2021). C2, C2 domain; p85B, p85-binding domain; rbd, Ras-binding domain; PI3Ka, PI3K accessory domain; PI3Kc, PI3K catalytic domain; PIK3CG_ABD, PIK3 catalytic subunit gamma adaptor-binding domain.

The catalytic subunits of Class I PI3K includes four highly homologous p110 isoforms (p110α,p110β, p110γ, and p110δ) encoded by genes PIK3CA, PIK3CB, PIK3CG, and PIK3CD, respectively (Zhao and Vogt, 2008). To investigate which subgroup the Djpi3k is classified, 30 genes encoding the p110 catalytic isoforms from 13 representative species were selected for phylogenetic analysis. As shown in Figure 1, all selected genes were mainly categorized into four clusters corresponding to PIK3CA (blue branch), PIK3CB (yellow branch), PIK3CG (green branch), and PIK3CD (red branch), among which Djpi3k (A0A222C681_DUGJA) was classified into the PIK3CA subgroup as well as another planarian ortholog (D2DJU4_SCHMD) from *Schmidtea mediterranea*. Domain annotations for each sequence were determined using the Pfam database (Mistry et al., 2021), and the predicted domains were consistent with the results from other databases (Supplementary Figure 1).

### Expression Pattern of *Djpi3k* During Planarian Regeneration

**FIGURE 2:**
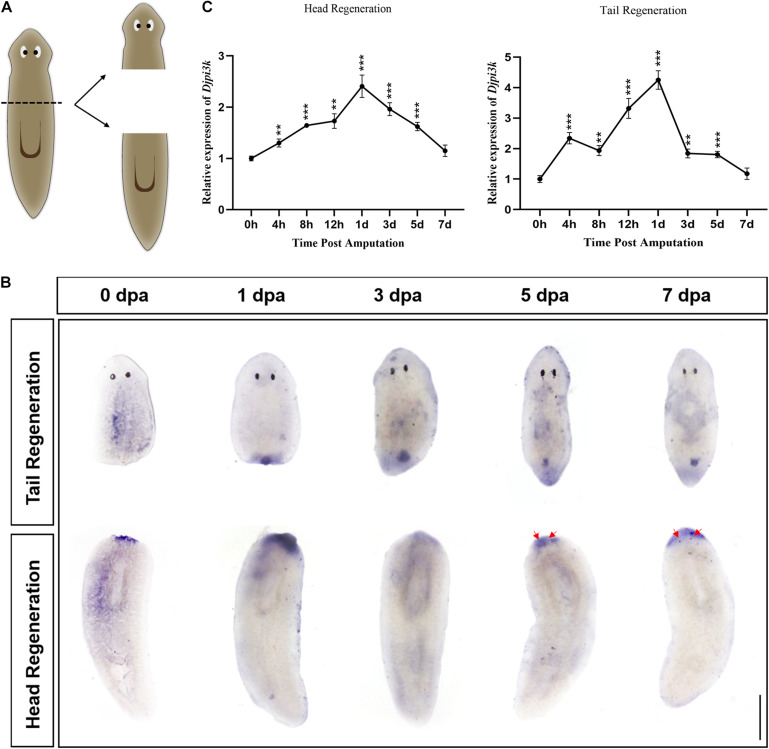
Expression pattern of *Djpi3k* during planarian regeneration. **(A)** Schematic representation of the general sampling set-up. **(B)** Spatiotemporal expression pattern of *Djpi3k* during planarian regeneration assessed by whole-mount *in situ* hybridization. Newly-regenerated photoreceptors are indicated with red arrows. Scale bar = 1 mm. **(C)** Fold change in mRNA expression levels of *Djpi3k* during planarian head and tail regeneration. All values are relative to the expression level of *Djpi3k* mRNA at 0 h. **P < 0.01 and ***P < 0.001, by the Student’s *t*-test.

To characterize the expression pattern of *Djpi3k* during planarian regeneration, animals were cut anterior of the pharynx and allowed to regenerate for 7 days (Figure 2A), samples at different regenerating time were fixed and subjected to WISH. The specification of the *Djpi3k* probe was confirmed in intact animals using a *Djpi3k* sense probe as a negative control and a neoblast marker gene *DjpiwiA* as a positive control (Supplementary Figures 2A,B). The WISH results showed that *Djpi3k* was expressed immediately after amputation at the wound surface, suggesting that the gene was in response to injury/tissue absence. High levels of *Djpi3k* expression were detected in the newly-regenerated blastema and pharynx-forming regions during regeneration. The *Djpi3k* level decreased gradually as the regeneration became complete (Figure 2B). The dynamic change of *Djpi3k* expression during planarian regeneration was also analyzed using qRT-PCR analysis during anterior and posterior regeneration (Figure 2C). The qRT-PCR results indicated that *Djpi3k* levels were upregulated following amputation, peaked at 1 day post amputation (dpa), and then fell to basal level until the regeneration was complete, which was consistent with the results of WISH analysis.

### Inhibition of PI3K Signaling Impairs Planarian Regeneration and Homeostasis

**FIGURE 3:**
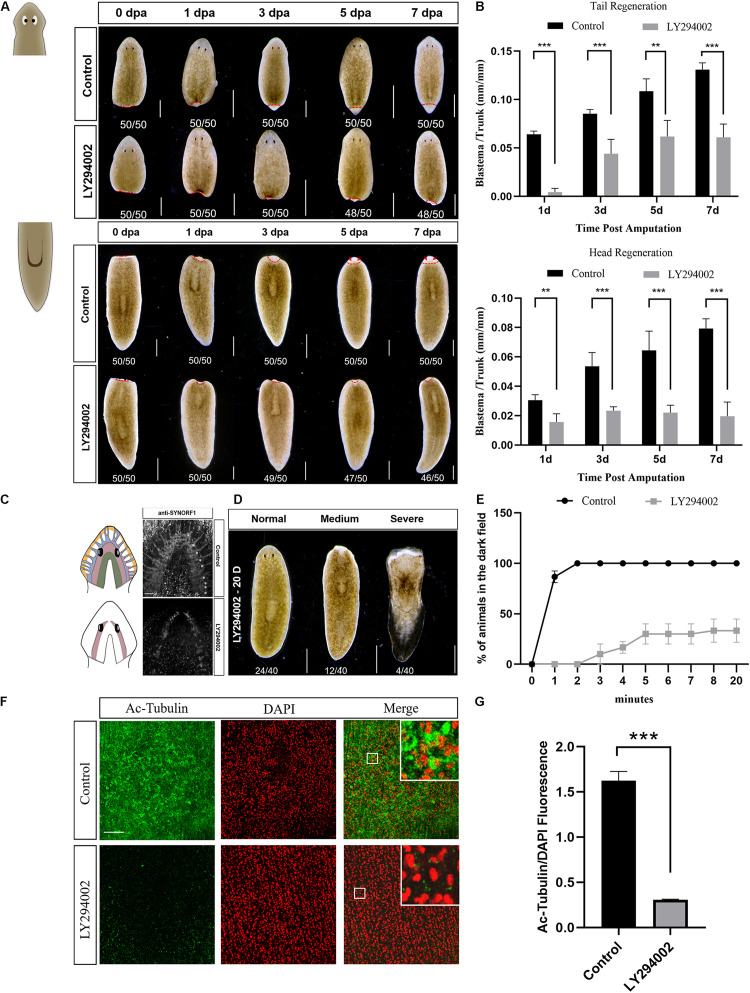
Effect of the PI3K inhibitor LY294002 on planarian regeneration and homeostasis. **(A)** Dorsal images of control and LY294002-treated head and tail fragments at 0, 1, 3, 5, and 7 days post amputation. Newly-regenerated photoreceptors are indicated with red arrows. Scale bar = 1 mm. **(B)** Quantification analysis of blastema growth. The length of the regenerated blastema was measured and normalized to the whole-body length of the trunk. **P < 0.01 and ***P < 0.001, by the Student’s *t*-test. **(C)** Cartoon and immunostaining of the regenerated CNS at 7 dpa using an anti-SYNORF1 antibody in control (top) and LY294002-treated animals (bottom). Lateral branch neurons are indicated with asterisks. Scale bar = 100 μm. **(D)** Representative phenotypes induced by a 20-day treatment of LY294002. Scale bar = 1 mm. **(E)** Planarian locomotion is hampered by LY294002 treatment. Gliding ability is evaluated by calculating the proportion of planarians that reach the dark zone on the side opposite to light. **(F)** Immunostaining of the ventral cilia using an anti-Ac-Tubulin antibody (green). DAPI is used to label the nucleus (red). Magnified images are shown in the right corner. Scale bar = 100 μm. **(G)** Quantification analysis of cilia density using the green/red fluorescence intensity ratio. ***P < 0.001, by the Student’s *t*-test.

To investigate the role of PI3K signaling in planarian regeneration, we inactivated the PI3K signaling by using LY294002, a selective PI3K inhibitor (Walker et al., 2000). Animals were amputated and exposed to a gradient concentration of LY294002 for 7 days. Treatment with 45 μM LY294002 caused damage to the epidermis followed soon by lysis, while animals survived by treatment with a moderate concentration at 30 μM (Supplementary Figure 3). However, both head and tail regeneration were largely impaired after 30 μM LY294002 treatment (Figure 3A). The LY294002 treated fragments only regrew marginal blastemas at 7 dpa, which were significantly smaller than those of the control animals (Figure 3B). In addition, no eyes were formed in the regenerating tail fragments at 7 dpa when the control animals had regenerated their eyes properly (Figure 3A). A similar inhibitory effect was observed in the animals treated with A66 (80 μM, Supplementary Figures 4A,B), a highly specific and selective p110α inhibitor (Jamieson et al., 2011). The planarian brain is mainly composed of four structurally and functionally different regions including mechanosensory neurons (yellow), lateral branch neurons (blue, nine asterisks), two main lobes (red), and photosensory neurons (green) (Agata and Umesono, 2008). We tested whether planarian brain regeneration was disturbed after treatment of LY294002 using an anti-SYNORF1 antibody that recognizes brain structures. The immunostaining results revealed that PI3K inhibition caused apparent defects in brain regeneration at 7 dpa, mainly manifested as incomplete main lobes, disappeared lateral branch neurons, and the brain commissure of the two lobes not connected in the top (Figure 3C). These data indicate that PI3K signaling is required for appropriate regeneration in planarians.

We next examined the effect of PI3K inhibition on planarian homeostasis, a self-regulating process allowing for tissue maintenance during adult cell turnover. After a 20-day treatment with LY294002, slight ulceration of the epidermis was observed in quite a few animals (12/40), and tissue lysis could be found in several animals (4/40) (Figure 3D). Although more than half of the animals appeared no morphological abnormality after LY294002 treatment, their locomotion was considerably hampered. When exposed to light, the control animals moved quickly away from the light and reached the dark field within 2 min. On the contrary, LY294002 treated animals twisted forward, flipped over, or stayed still during their traveling toward the dark field. No animals reached the dark field until 3 min post light exposure, and two-third of the animals never arrived at the dark field even at 20 min after light exposure (Figure 3E and Supplementary Videos 1, 2). Plane gliding is mediated by synchronous cilia movement on the ventral surface of planarians (Rink et al., 2009). To verify the possibility that LY294002-induced impaired gliding ability was caused by cilia defect, we used whole-mount staining with the anti-Ac-tubulin antibody to visualize the integrity of ciliary epithelial cells in the ventral epithelium. The LY294002 treated animals displayed poor staining in cilia that were severely shortened, whereas the control animals showed dense coverage of cilia (Figures 3F,G). Our results suggest that impaired motility in the *Djpi3k* inhibition phenotype is most likely due to insufficient density of ventral epithelial cells. Thus, these results indicated that *PI3K* activation is required for planarian homeostasis, especially cilia maintenance.

**FIGURE 4:**
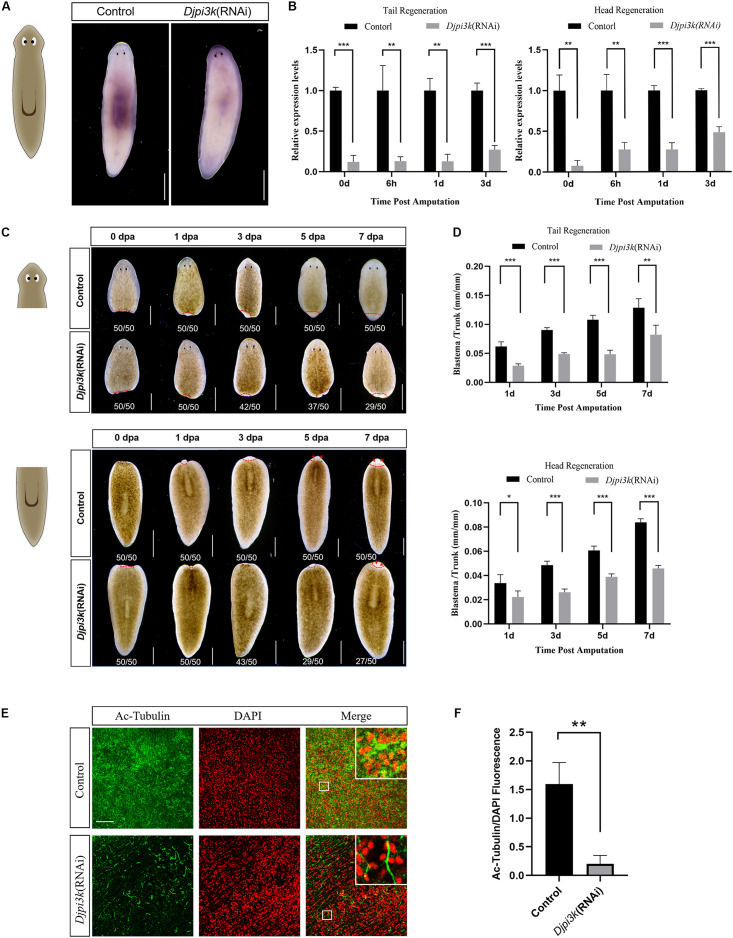
Effect of *Djpi3k* RNAi knockdown on planarian regeneration and homeostasis. **(A)** RNAi efficiency was verified in intact animals by WISH using an antisense *Djpi3k* probe. Scale bar = 1 mm. **(B)** RNAi efficiency was examined during planarian regeneration by qRT-PCR assay. **P < 0.01 and ***P < 0.001, by the Student’s *t*-test. **(C)** Dorsal images of regenerating trunk fragments from the control and *Djpi3k* (RNAi) animals at 0, 1, 3, 5, and 7 days post amputation. Newly-regenerated photoreceptors are indicated with red arrows. Scale bar = 1 mm. **(D)** Quantification analysis of blastema growth. The length of the regenerated blastema was measured and normalized to the whole-body length of the trunk. *P < 0.05, **P < 0.01, and ***P < 0.001, by the Student’s *t*-test. **(E)** Immunostaining of the ventral cilia using an anti-Ac-Tubulin antibody (green). DAPI is used to label the nucleus (red). Magnified images are shown in the right corner. Scale bar = 100 μm. **(F)** Quantification analysis of cilia density using the green/red fluorescence intensity ratio. **P < 0.01, by the Student’s *t*-test.

RNAi has been widely used to study regeneration-associated genes in planarians (Sánchez Alvarado and Newmark, 1999; Newmark et al., 2003; Rouhana et al., 2013). A bacterial feeding protocol (Newmark et al., 2003; Zeng et al., 2013) was employed in this study to investigate the role of *Djpi3k* in planarian regeneration and homeostasis. RNAi efficiency was confirmed by WISH and qRT-PCR analysis (Figures 4A,B). *Djpi3k*(RNAi) animals also exhibited limited regenerative capacity compared with the control animals (Figures 4C,D). Different from the LY294002 treatment, RNAi-mediated PI3K inhibition led to relatively mild phenotypes and a lower proportion of animals with regeneration defects (27/50 vs 46/50 in head regeneration, 29/50 vs 48/50 in tail regeneration). A similar situation occurs in tissue homeostasis, decreased cilia density was observed in *Djpi3k*(RNAi) animals, nevertheless, the integrity of the ciliated structure was maintained (Figures 4E,F). Taken together, these data suggest that PI3K signaling is required for planarian regeneration and tissue maintenance.

### PI3K Regulates Mitotic and Apoptotic Responses During Planarian Regeneration

**FIGURE 5:**
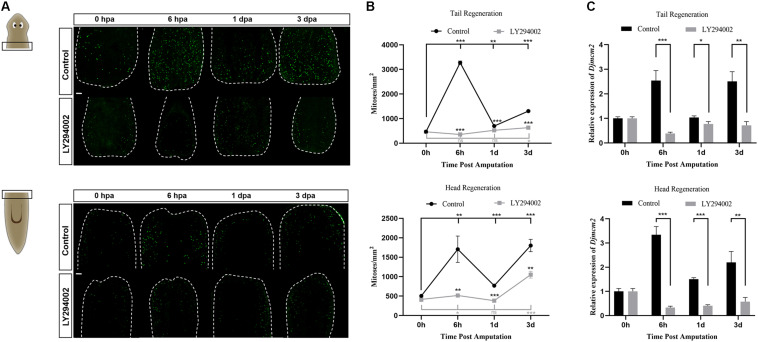
Effect of the PI3K inhibitor LY294002 on neoblast proliferation during planarian regeneration. **(A)** Representative images showing mitotic activity (green dots, H3P positive cells) at 0 h, 6 h, 1 days, and 3 days post amputation. Scale bar = 100 μm. **(B)** Quantification of the average number of H3P positive cells/mm^2^ in the regenerating trunk fragments at indicated time point. *P < 0.05, **P < 0.01, ***P < 0.001, and ns, not significant, by the Student’s *t*-test. **(C)** Relative expression levels of *Djmcm2* gene in regenerating head and tail fragments. The expression level of *Djmcm2* at 0 hpa was set to one. *P < 0.05, **P < 0.01, and ***P < 0.001, by the Student’s *t*-test.

PI3K signaling participates in multiple biological processes associated with cell growth and proliferation (Madsen, 2020). Neoblasts represent the only proliferative cell population in planarians and are therefore the source of new cells for normal tissue turnover, growth, and regeneration (Wagner et al., 2011). We checked the effect of PI3K inhibition on the mitotic activity of neoblasts during planarian regeneration using the anti-H3P antibody, which labels cells in the G2/M phase (Peiris et al., 2012, 2016). In intact planarians, the H3P-positive cells were detected distributed throughout the body except in the head region anterior to the eyes and the pharynx region (Supplementary Figure 2C), which was consistent with the distribution patterns of planarian neoblasts reported in previous studies (Newmark and Sánchez Alvarado, 2000; Salvetti et al., 2000) and further verified the specificity of the H3P staining results in our study. For regeneration studies, the number of the H3P-positive cells in the control animals reached the first peak of mitosis at 6 hpa and the second peak at 3 dpa, which was consistent with previous studies (Wenemoser and Reddien, 2010). However, the number of the H3P-positive cells was dramatically decreased after LY294002 treatment, making the mitotic response vanished at the early stage of planarian regeneration (Figures 5A,B). Knockdown of *Djpi3k* also reduced the neoblast number, the mitotic response of *Djpi3k*(RNAi) animals was alleviated but still existed (Supplementary Figures 5A,B). Moreover, the expression pattern of *Djmcm2*, a neoblast marker (Salvetti et al., 2000), was evaluated before and after PI3K inactivation. The qRT-PCR results showed that the expression levels of *Djmcm2* could reflect the mitotic trends as manifested by H3P staining, and were substantially suppressed after LY294002/A66 treatment or *Djpi3k* knockdown (Figure 5C and Supplementary Figures 4C, 5C). Our results suggest that PI3K signaling is required to maintain the appropriate number of proliferating neoblast during tissue renewal in adult planarians.

**FIGURE 6:**
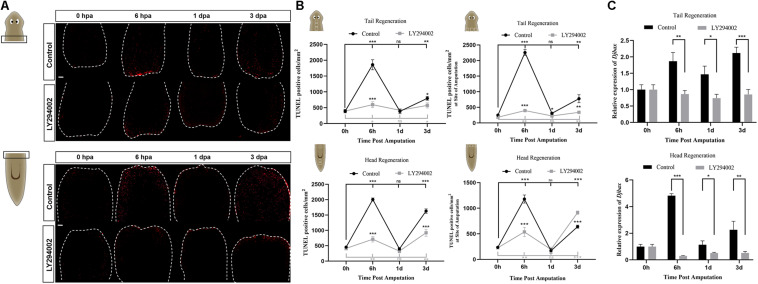
Effect of the PI3K inhibitor LY294002 on apoptotic cell death during planarian regeneration. **(A)** Immunostaining of TUNEL-positive nuclei of trunk fragments at 0 h, 6 h, 1 days, and 3 days post amputation. Scale bar = 100 μm. **(B)** Quantification of the average number of TUNEL-positive cells/mm^2^ in the whole trunk fragments (left panel) or at the amputation sites (right). *P < 0.05, **P < 0.01, ***P < 0.001, and ns, not significant, by the Student’s *t*-test. **(C)** Relative expression levels of *Djbax* gene in regenerating head and tail fragments. The expression level of *Djbax* at 0 hpa was set to one. *P < 0.05, **P < 0.01, ***P < 0.001, and ns, not significant, by the Student’s *t*-test.

Apoptosis, a prominent form of cell death in planarians, facilitates cell turnover during homeostasis and regeneration (Pellettieri et al., 2010). To investigate the possible role of PI3K signaling in apoptotic cell death during planarian regeneration, the spatiotemporal distribution of apoptosis was evaluated by TUNEL staining. The results revealed that the number of apoptotic cells in both the head and tail fragments was substantially decreased throughout the regeneration period after LY294002 treatment (Figure 6A). The system-wide cell death expected at 3 dpa was also suppressed by LY294002 treatment (Figure 6A and left panel of Figure 6B). It was noted that the localized cell death response at 6 hpa in control animals was also largely impaired by LY294002 treatment (Figure 6B, right panel). Similarly, cell death in *Djpi3k*(RNAi) animals failed to localize at the amputation site as is expected at 6 hpa (Supplementary Figures 6A,B). Nevertheless, *Djpi3k*(RNAi) animals showed a delayed apoptotic peak at 1 dpa during head and tail regeneration. Moreover, the expression pattern of *Djbax*, a pro-apoptotic marker gene, was evaluated by qRT-PCR before and after PI3K inhibition, and the results were consistent with the TUNEL staining results (Figure 6C and Supplementary Figures 4D, 6C). These findings indicate that PI3K signaling is essential for controlling wound-induced apoptotic cell death during tissue regeneration. Taken together, we propose *Djpi3k* functions as a regulator of proliferation and apoptosis during large-scale tissue regeneration in planarians.

## Discussion

PI3K signaling is one of the most important and conserved signaling pathways in eukaryotic cells and is closely related to animal regeneration (Chen et al., 2013). In the present study, the planarian *Dugesia japonica* was employed to study the potential role of PI3K signaling in the regulation of the regeneration process. Our results demonstrated that PI3K plays a crucial role in maintaining proper mitotic and apoptotic responses to amputation, which might be essential for planarian regeneration.

According to sequence homology and substrate specificity, PI3Ks are mainly divided into three categories: Class I, II, and III (Thorpe et al., 2015). The catalytic subunits of Class I PI3K includes four highly homologous p110 isoforms (p110α,p110β, p110γ, and p110δ) encoded by genes PIK3CA, PIK3CB, PIK3CG, and PIK3CD, respectively (Zhao and Vogt, 2008). The results of conserved domain prediction and phylogenetic analysis suggested *Djpi3k* is highly homologous to the *PIK3CA* gene, which encodes the best-characterized Class I PI3K catalytic isoform p110α. The ubiquitously expressed p110α is crucial for organismal growth and development, with pleiotropic functions including self-renewal of stem cells, tissue maintenance, and metabolic regulation (Madsen, 2020). Our phylogenetic results demonstrate that the majority of the PI3K catalytic isoforms in each subgroup share similarities in sequence homology and conserved domain pattern. Nevertheless, the PI3K-like genes derived from *Caenorhabditis elegans* (AGE1_CAEEL) and *Leishmania infantum* (A4HVU4_LEIIN), lacking the agonist binding domain (ABD) though, were also clustered into the PIK3CG subgroup probably due to the sequence homology. Surprisingly, the hydra and arthropod PI3K-like genes were not clustered into any of the four sub-categories, probably because of the low similarity in domain pattern and sequence homology. Interestingly, the amphibian *Xenopus tropicalis* does not contain a p110 delta isoform, which is quite different from other chordates possessing four p110 isoforms. These findings suggest that the combined utilization of phylogenetic analysis and associated annotations provide a powerful tool to organize genes of interest into sub-categories.

The precise modulation of PI3K signaling is critical for the growth and development of the organism, while uncontrolled activation of PI3K leads to pathological phenomena including tumorigenesis (Lien et al., 2017). In this study, the expression pattern of *Djpi3k* was characterized and suggests it appears to be an early wound response gene. Wound-induced genes, including a *Schmidtea mediterranea pi3k* like gene, have been well described in planarians (Wenemoser et al., 2012) and clustered based on their onset and offset times into three groups: *early*, *late*, and *sustained* (Wurtzel et al., 2015). The *early* cluster genes, such as *egr-l 1*, *ston*, and *heat shock protein genes*, are immediately induced post amputation and fast to decay. Conversely, the *late* and *sustained* cluster genes, such as *runt-1* and *inhibin-1*, also exhibit rapid induction but last for days, which resembles the expression pattern of *Djpi3k* characterized in this study. It is now well accepted that a generic response triggered by different types of injuries leads to mere wound healing or regeneration initiation depending on if tissue loss occurs (Owlarn et al., 2017). During planarian regeneration, *Djpi3k* is highly expressed in newly-regenerated blastema and pharynx-forming regions, implying that it can somehow sense the tissue context. Further research should be undertaken to investigate what signals the *Djpi3k* expression is triggered by and how PI3K signaling translates the generic wound into regeneration.

To investigate the role of PI3K signaling in planarian regeneration and homeostasis, a loss-of-function study was first conducted using small molecules. LY294002 treatment leads to regeneration defects in planarians manifested as regrowing marginal blastemas and incomplete brain structures. Tissue homeostasis is also disturbed by long-term treatment of LY294002, one of the hallmarks is the impaired cilia-dependent gliding ability. In accordance with the present results, previous studies have demonstrated that other components of the PI3K pathway play crucial roles in planarian regeneration and tissue maintenance. Akt, as a main downstream target of PI3K, regulates neoblast biology and cell death response during tissue repair in planarian *Schmidtea mediterranea*. Knockdown of *Smed-Akt* also impairs the maintenance of adult tissues including the nervous and excretory systems and ciliated structures (Peiris et al., 2016). Similarly, systemic inhibition of Target of Rapamycin (TOR) signaling leads to restricted proliferative capacity of neoblasts and tissue degeneration. Intriguingly, though blastema formation is hampered by *Smed-TOR*(RNAi), regeneration of CNS and visual neurons takes place within pre-existing tissue (Peiris et al., 2012). On the contrary, a phosphoinositide 3-kinase-related kinase (PIKK) family member SMG-1 acts antagonistically with mTOR signaling and works as a brake on the initial response to injury. Loss of SMG-1 leads to hyperactive responses to injury and subsequent growth that continues out of control (González-Estévez et al., 2012). Tumor suppressor gene *PTEN* is a predominant negative regulator of PI3K signaling (Song et al., 2012). The hallmarks of RNAi phenotype after silencing of *Smed-PTEN-1* and *Smed-PTEN-2* are hyperproliferation of neoblasts, tissue disorganization, and a significant accumulation of postmitotic cells with impaired differentiation capacity (Oviedo et al., 2008), the latter of which also accords with the earlier observations in Pten-ablated mouse embryonic stem cells (Di Cristofano et al., 1998). These findings suggest that PI3K signaling is required for planarian regeneration and tissue homeostasis.

During planarian regeneration, neoblasts undergo extensive cell division to regenerate lost or damaged tissue (Wenemoser and Reddien, 2010), and apoptotic cell death coordinates with cell division to drive adult tissue renewal and repair (Pellettieri et al., 2010). Thus, we further checked the effect of PI3K inhibition on cell proliferation and apoptotic cell death during planarian regeneration. The mitotic and apoptotic responses to amputation are substantially abated throughout the regeneration period after LY294002 or A66 treatment, which may contribute to the inhibitor-induced regeneration defects. To verify the specificity of these small molecules, RNAi experiments were also conducted. One interesting finding is that *Djpi3k*(RNAi) animals exhibit similar but relatively mild phenotypes in morphology compared with the inhibitor-treated animals. For neoblast proliferation, LY294002 completely vanishes planarian mitotic response to amputation at the early regenerative stage (Figure 5B), while a weak but significant mitotic response still exists in the regenerating *Djpi3k*(RNAi) animals (Supplementary Figure 5B). For cell death, it is unexpectedly to note that LY294002 acts to “blunt” planarian apoptotic response while knockdown of *Djpi3k* acts more likely to “postpone” the onset of the first wave of apoptotic cell death. These findings suggest that the different phenotypes between PI3K inhibitor-treated animals and *Djpi3k*(RNAi) animals may be attributed to the different extent of inhibition against PI3K activity.

**FIGURE 7:**
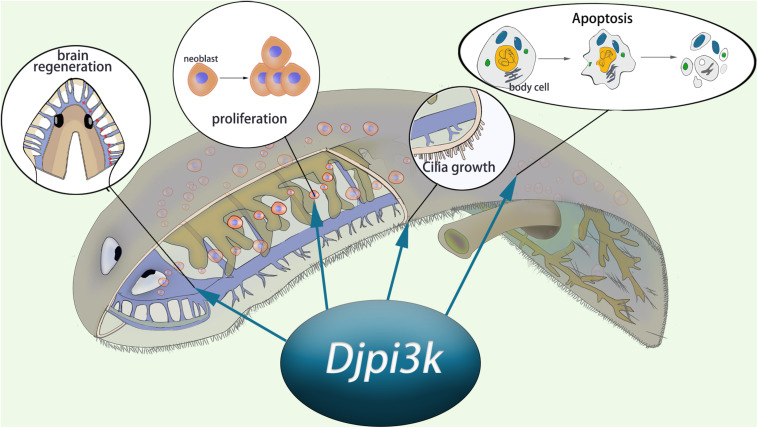
Schematic representation of the essential role of *Djpi3k* in planarian regeneration and tissue maintenance.

Taken together, we propose that PI3K signaling is required for planarian regeneration and homeostasis (Figure 7). A loss-of-function assay using small molecules or RNAi technique suggests PI3K plays crucial roles in tissue regeneration, cilia maintenance, neoblast proliferation, and apoptotic cell death. Our findings complement those of earlier studies relative to the planarian PI3K signaling pathway and provide insights for investigating the disease-related genes in the regeneration-competent organism *in vivo*.

## Data Availability Statement

The original contributions presented in the study are included in the article/Supplementary Material, further inquiries can be directed to the corresponding author/s.

## Author Contributions

YY and FC designed and supervised the study. HZ, HL, LL, and GY performed the experiments. QX, WW, and XW analyzed the data. YY and HZ wrote the manuscript. All authors contributed to the article and approved the submitted version.

## Conflict of Interest

The authors declare that the research was conducted in the absence of any commercial or financial relationships that could be construed as a potential conflict of interest.

## Publisher’s Note

All claims expressed in this article are solely those of the authors and do not necessarily represent those of their affiliated organizations, or those of the publisher, the editors and the reviewers. Any product that may be evaluated in this article, or claim that may be made by its manufacturer, is not guaranteed or endorsed by the publisher.

## Footnotes

1
https://www.expasy.org/resources/protparam

## Supplementary Material

Supplementary Figure 1 Conserved domain prediction of Djpi3k protein. The conserved domains of Djpi3k protein were predicted using the SMART database (A) and the NCBI Conserved Domain Database (B). The predicted Class I PI3K catalytic domain and accessory domain were labeled with red stars.

Supplementary Figure 2 Specificity verification of the WISH and H3P immunostaining analyses in intact animals. Whole-mount *in situ* hybridization with sense and anti-sense *Djpi3k* (A) or *DjpiwiA* (B) probes, respectively. Scale bar = 1 mm. (C) Distribution of proliferative neoblasts in an intact planarian detected by immunostaining using an anti-H3P antibody. Photoreceptors are indicated with white stars and the pharynx region is outlined with white dashed lines. Scale bar = 200 μm.

Supplementary Figure 3 Representative regenerative phenotypes induced by a 7-day treatment of a concentration gradient of LY2940027. Scale bar = 1 mm.

Supplementary Figure 4 Effect of the PI3K inhibitor A66 on planarian regeneration. **(A)** Dorsal images of control and A66-treated head and tail fragments at 0, 1, 3, 5, and 7 days post amputation. Newly-regenerated photoreceptors are indicated with red arrows. Scale bar = 1 mm. **(B)** Quantification analysis of blastema growth. The length of the regenerated blastema was measured and normalized to the whole-body length of the trunk. ^∗^*P* < 0.05 and ^∗∗∗^*P* < 0.001, by the Student’s *t*-test. **(C,D)** Effect of the PI3K inhibitor A66 on *Djmcm2* **(C)** and *Djbax* **(D)** expression during planarian regeneration. The expression level of *Djmcm2* or *Djbax* at 0 hpa was set to one. ^∗^*P* < 0.05, ^∗∗^*P* < 0.01, ^∗∗∗^*P* < 0.001, and ns, not significant, by the Student’s *t*-test.

Supplementary Figure 5 Effect of *Djpi3k* RNAi knockdown on neoblast proliferation during planarian regeneration. **(A)** Representative images showing mitotic activity (green dots, H3P positive cells) at 0 h, 6 h, 1 day, and 3 days post amputation. Scale bar = 100 μm. **(B)** Quantification of the average number of H3P positive cells/mm^2^ in the regenerating trunk fragments at indicated time point. ^∗∗^*P* < 0.01, ^∗∗∗^*P* < 0.001, and ns, not significant, by the Student’s *t*-test. **(C)** Relative expression levels of *Djmcm2* gene in regenerating head and tail fragments. The expression level of *Djmcm2* at 0 hpa was set to one. ^∗∗^*P* < 0.01 and ^∗∗∗^*P* < 0.001, by the Student’s *t*-test.

Supplementary Figure 6 Effect of *Djpi3k* RNAi knockdown on apoptotic cell death during planarian regeneration. **(A)** Immunostaining of TUNEL-positive nuclei of trunk fragments at 0 h, 6 h, 1 days, and 3 days post amputation. Scale bar = 100 μm. **(B)** Quantification of the average number of TUNEL-positive cells/mm^2^ in the whole trunk fragments (left panel) or at the amputation sites (right). ^∗^*P* < 0.05, ^∗∗^*P* < 0.01, ^∗∗∗^*P* < 0.001, and ns, not significant, by the Student’s *t*-test. **(C)** Relative expression levels of *Djbax* gene in regenerating head and tail fragments. The expression level of *Djbax* at 0 hpa was set to one. ^∗∗^*P* < 0.01, ^∗∗∗^*P* < 0.001, and ns, not significant, by the Student’s *t*-test.

Supplementary Video 1 Representative video showing the gliding ability of control animals. All control animals moved quickly away from the light and reached the dark field within 2 min when exposed to light.

Supplementary Video 2 Representative video showing the gliding ability of LY294002-treated animals. Two-third of the LY294002-treated animals never arrived at the dark field even at 20 min post light exposure, exhibiting photophobic but impaired locomotion behaviors including twisting, flipping, and staying still.

## Abbreviations

*Dj*: *Dugesia japonica*
PI3K: phosphatidylinositol 3-kinase
dsRNA: double-stranded RNA
RNAi: RNA interference
ORF: open reading frame
pI: isoelectric point
WISH: whole-mount *in situ* hybridization
qRT-PCR: quantitative Real-time PCR
hpa/dpa: hours/days post amputation
H3P: phosphorylated histone H3
TUNEL: terminal deoxynucleotidyl transferase dUTP nick end labeling.
