# Supplementary figures and images for: PI3K Plays an Essential Role in Planarian Regeneration and Tissue Maintenance
Source: Front Cell Dev Biol. 2021 Aug 6;9:649656. doi: 10.3389/fcell.2021.649656 (PMC8377419; doi:10.3389/fcell.2021.649656)

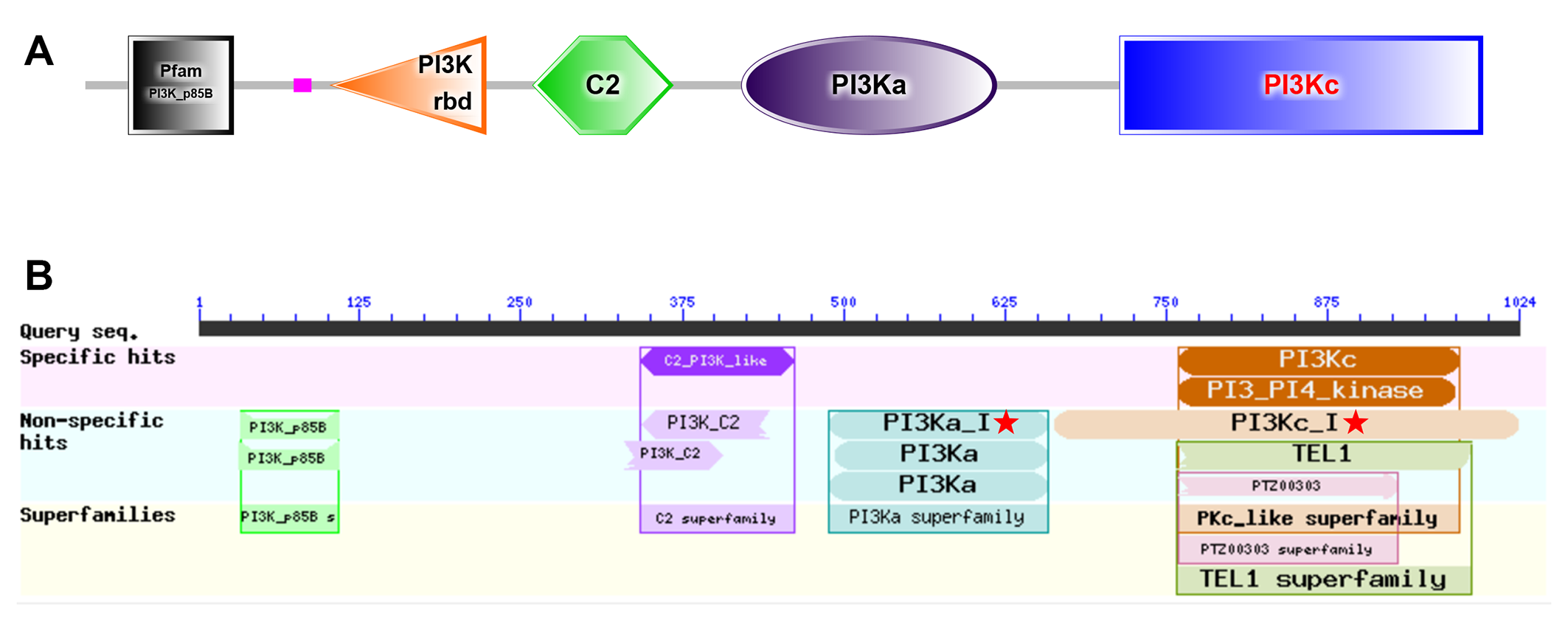

Supplement: Supplementary Figure 1 — Conserved domain prediction of Djpi3k protein. The conserved domains of Djpi3k protein were predicted using the SMART database (A) and the NCBI Conserved Domain Database (B). The predicted Class I PI3K catalytic domain and accessory domain were labeled with red stars. [file Image_1.TIF]

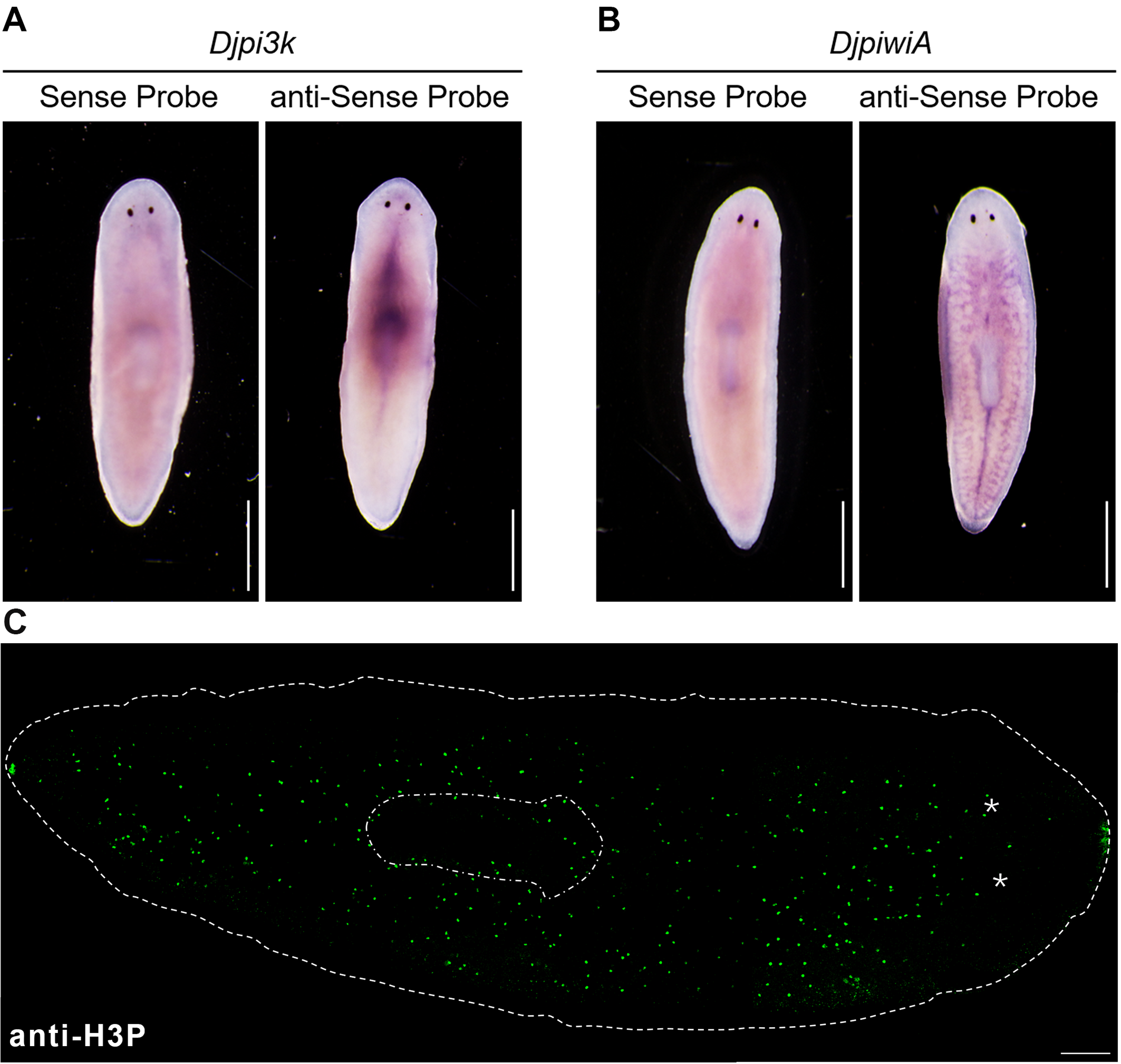

Supplement: Supplementary Figure 2 — Specificity verification of the WISH and H3P immunostaining analyses in intact animals. Whole-mount in situ hybridization with sense and anti-sense Djpi3k (A) or DjpiwiA (B) probes, respectively. Scale bar = 1 mm. (C) Distribution of proliferative neoblasts in an intact planarian detected by immunostaining using an anti-H3P antibody. Photoreceptors are indicated with white stars and the pharynx region is outlined with white dashed lines. Scale bar = 200 μm. [file Image_2.TIF]

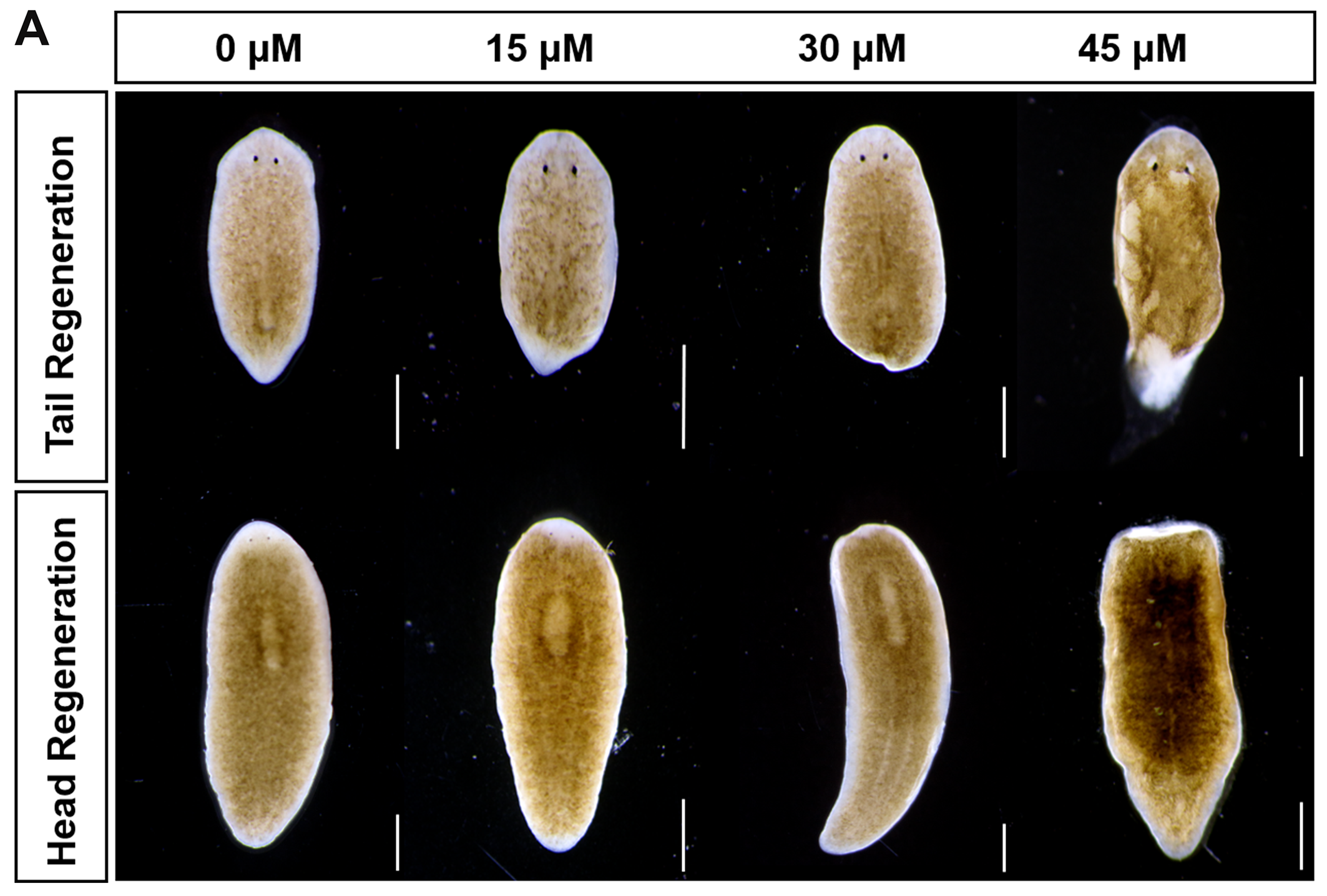

Supplement: Supplementary Figure 3 — Representative regenerative phenotypes induced by a 7-day treatment of a concentration gradient of LY2940027. Scale bar = 1 mm. [file Image_3.TIF]

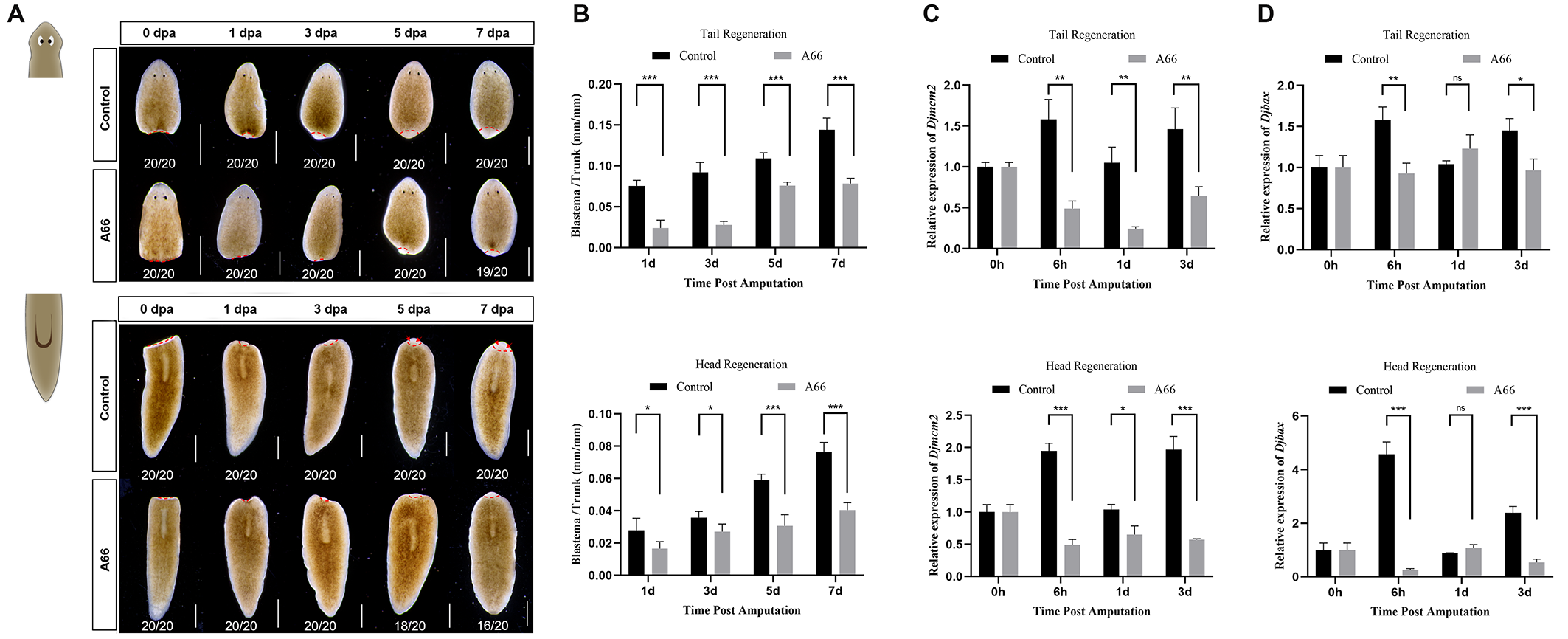

Supplement: Supplementary Figure 4 — Effect of the PI3K inhibitor A66 on planarian regeneration. (A) Dorsal images of control and A66-treated head and tail fragments at 0, 1, 3, 5, and 7 days post amputation. Newly-regenerated photoreceptors are indicated with red arrows. Scale bar = 1 mm. (B) Quantification analysis of blastema growth. The length of the regenerated blastema was measured and normalized to the whole-body length of the trunk. ∗P < 0.05 and ∗∗∗P < 0.001, by the Student’s t-test. (C,D) Effect of the PI3K inhibitor A66 on Djmcm2 (C) and Djbax (D) expression during planarian regeneration. The expression level of Djmcm2 or Djbax at 0 hpa was set to one. ∗P < 0.05, ∗∗P < 0.01, ∗∗∗P < 0.001, and ns, not significant, by the Student’s t-test. [file Image_4.TIF]

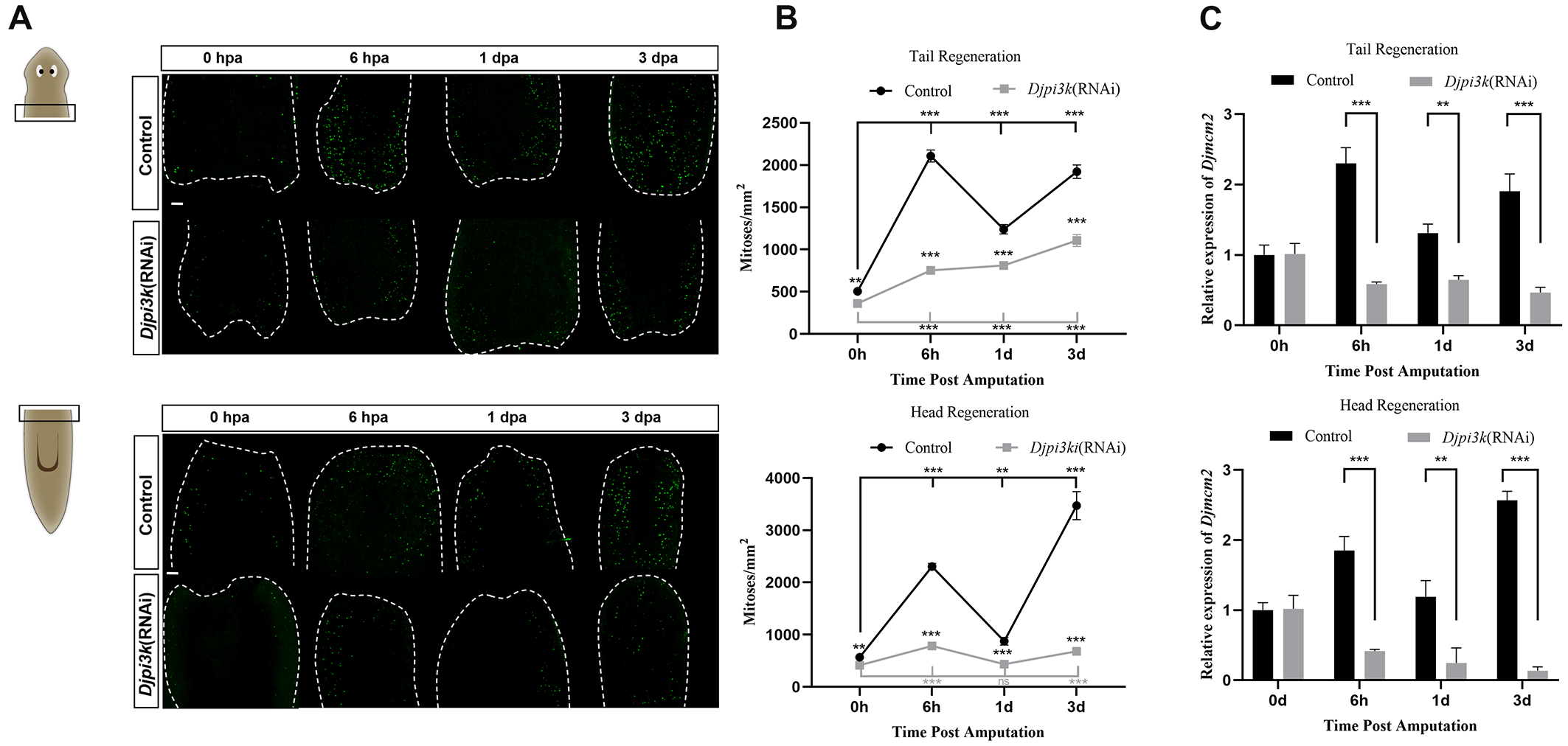

Supplement: Supplementary Figure 5 — Effect of Djpi3k RNAi knockdown on neoblast proliferation during planarian regeneration. (A) Representative images showing mitotic activity (green dots, H3P positive cells) at 0 h, 6 h, 1 day, and 3 days post amputation. Scale bar = 100 μm. (B) Quantification of the average number of H3P positive cells/mm2 in the regenerating trunk fragments at indicated time point. ∗∗P < 0.01, ∗∗∗P < 0.001, and ns, not significant, by the Student’s t-test. (C) Relative expression levels of Djmcm2 gene in regenerating head and tail fragments. The expression level of Djmcm2 at 0 hpa was set to one. ∗∗P < 0.01 and ∗∗∗P < 0.001, by the Student’s t-test. [file Image_5.TIF]

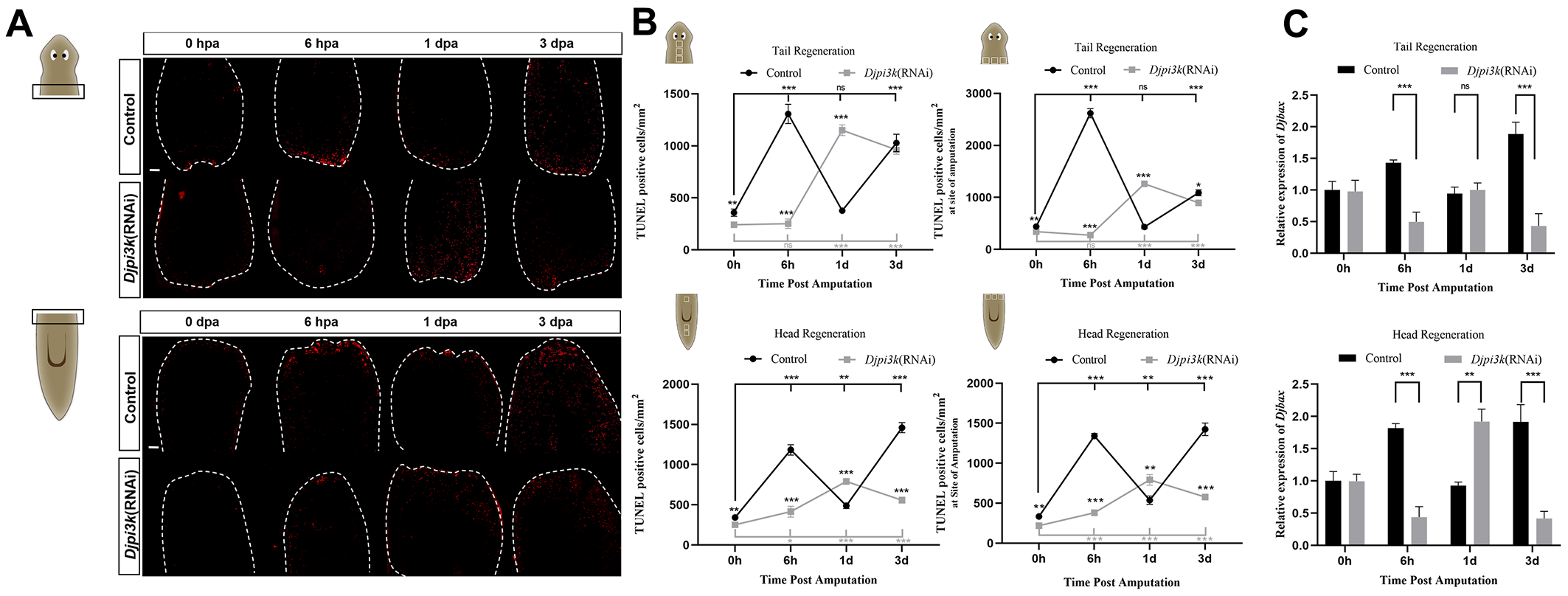

Supplement: Supplementary Figure 6 — Effect of Djpi3k RNAi knockdown on apoptotic cell death during planarian regeneration. (A) Immunostaining of TUNEL-positive nuclei of trunk fragments at 0 h, 6 h, 1 days, and 3 days post amputation. Scale bar = 100 μm. (B) Quantification of the average number of TUNEL-positive cells/mm2 in the whole trunk fragments (left panel) or at the amputation sites (right). ∗P < 0.05, ∗∗P < 0.01, ∗∗∗P < 0.001, and ns, not significant, by the Student’s t-test. (C) Relative expression levels of Djbax gene in regenerating head and tail fragments. The expression level of Djbax at 0 hpa was set to one. ∗∗P < 0.01, ∗∗∗P < 0.001, and ns, not significant, by the Student’s t-test. [file Image_6.TIF]
