# Supplementary material for: PI3K Plays an Essential Role in Planarian Regeneration and Tissue Maintenance
Source: Front Cell Dev Biol. 2021 Aug 6;9:649656. doi: 10.3389/fcell.2021.649656 (PMC8377419; doi:10.3389/fcell.2021.649656)
Supplement: Supplementary file 9 [file Table_1.DOCX]

**Table S1. PCR primers used in this study**

| **Primer name** | **Sequence (5’-3’)** | **Purpose** |
| --- | --- | --- |
| Djpi3k-RNAi-F1 | CTAGCTAGCTAGTTCCAAGCGACCATC | RNAi |
| Djpi3k-RNAi-R1 | CCGCTCGAGAACAGCAAACCGTCTCAA | RNAi |
| Djpi3k-RNAi-F2 | CTAGCTAGCGGATGCCTTGCTTCTGG | RNAi |
| Djpi3k-RNAi-R2 | CCGCTCGAGAACAGCAAACCGTCTCAA | RNAi |
| Djpi3k-probe-F | GGAGATGATTTGAGACAGG | Probe synthesis |
| Djpi3k-probe-R | CAGCCAATGAGACAAC | Probe synthesis |
| Djpi3k-QF | TCTTCATACATATTCTGTGGCGTA | qRT-PCR |
| Djpi3k-QR | ACAAAGAAGTTGTGATTCATCG | qRT-PCR |
| DjACTB-QF | GTATGCATCGGGTCGAACAA | qRT-PCR |
| DjACTB-QR | GTGGAAGAGCGTAACCTTCATAG | qRT-PCR |
| Djmcm2-QF | GGAGAAGAAGGAAGTGGTGAAA | qRT-PCR |
| Djmcm2-QR | CTCGCTGTCTCGCGTAAAT | qRT-PCR |
| Djbax-QF | ATGGTGGCTGGGAAGGC | qRT-PCR |
| Djbax-QR | ACTCGTGCAATTCTCGACAGAT | qRT-PCR |
